# A role for planar cell polarity during early endoderm morphogenesis

**DOI:** 10.1242/bio.021899

**Published:** 2017-04-10

**Authors:** Lee B. Miles, Takamasa Mizoguchi, Yutaka Kikuchi, Heather Verkade

**Affiliations:** 1 School of Biological Sciences, Monash University, Clayton, Victoria 3800, Australia; 2 Graduate School of Pharmaceutical sciences, Chiba University, Chuo-ku 260-8675, Japan; 3 Department of Biological Science, Graduate School of Science, Hiroshima University, Higashi-Hiroshima, Hiroshima 739-8526, Japan

**Keywords:** Endoderm, Planar cell polarity (PCP), Zebrafish, Midline aggregation, Development

## Abstract

The zebrafish endoderm begins to develop at gastrulation stages as a monolayer of cells. The behaviour of the endoderm during gastrulation stages is well understood. However, knowledge of the morphogenic movements of the endoderm during somitogenesis stages, as it forms a mesenchymal rod, is lacking. Here we characterise endodermal development during somitogenesis stages, and describe the morphogenic movements as the endoderm transitions from a monolayer of cells into a mesenchymal endodermal rod. We demonstrate that, unlike the overlying mesoderm, endodermal cells are not polarised during their migration to the midline at early somitogenesis stages. Specifically, we describe the stage at which endodermal cells begin to leave the monolayer, a process we have termed ‘midline aggregation’. The planar cell polarity (PCP) signalling pathway is known to regulate mesodermal and ectodermal cell convergence towards the dorsal midline. However, a role for PCP signalling in endoderm migration to the midline during somitogenesis stages has not been established. In this report, we investigate the role for PCP signalling in multiple phases of endoderm development during somitogenesis stages. Our data exclude involvement of PCP signalling in endodermal cells as they leave the monolayer.

## Abstract (precis)

**Summary:** Here we characterise endodermal morphogenesis during somitogenesis stages, and show that PCP signalling plays a mixed role in endoderm development during these stages.

## INTRODUCTION

During early development, the zebrafish endoderm undergoes a number of distinct morphogenetic stages to generate the gut and associated organs. The stages that have been characterised are: specification of the endoderm, migration of the endoderm during early gastrulation, formation of the endodermal rod, and organogenesis stages. However, the exact movements of the endoderm between mesoderm-dependent midline migration and during the formation of the endodermal rod have not been examined in detail. This study aims to fill that knowledge gap.

**Fig. 1.**
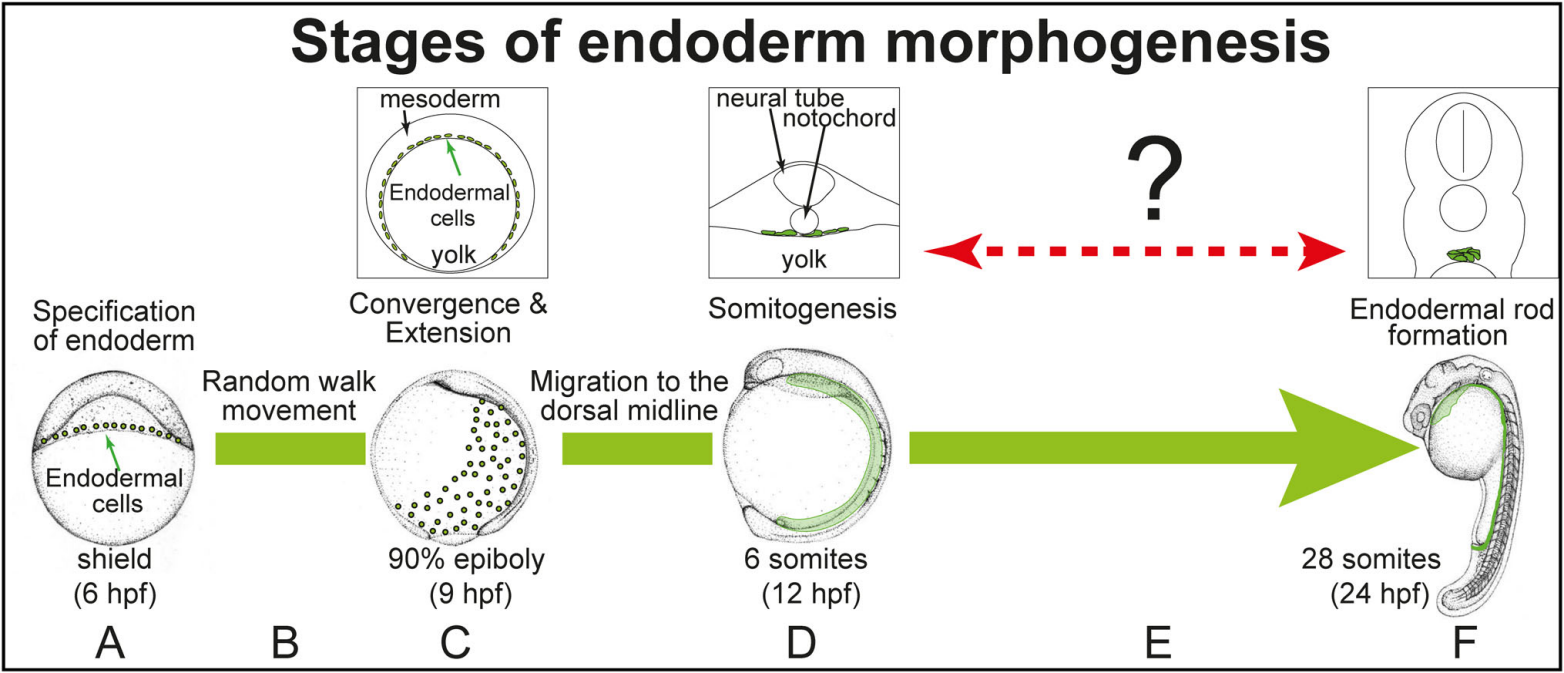
**Stages of endodermal morphogenesis.** (A) The endoderm is specified from a subset of cells in contact with the yolk syncytial layer (YSL) at shield. (B) Between shield and 90% epiboly the endoderm undergoes a random walk to spread out over the yolk. (C) At the onset of convergence and extension (C&E) (75% epiboly) the endoderm starts to migrate to the dorsal midline. (D) At early-somitogenesis stages the endoderm forms two broad stripes either side of the dorsal midline, running the antero-posterior length of the trunk of the embryo. (E) At an unknown stage during somitogenesis the endoderm undergoes a morphogenic change to form a primitive mesenchymal endodermal rod, which is complete by 28 somites (24 hpf) (F). The green dots or green shaded areas represent the endoderm. Inset are representations to show the locations of the endoderm in embryo cross-sections. Modified from Kimmel et al. (1995).

Zebrafish endoderm is specified during gastrulation from a subset of cells that have involuted and are now thus closely associated with the yolk (Fig. 1A) (Alexander and Stainier, 1999). These cells are positioned between the yolk and the mesoderm and migrate in all directions as a dispersed monolayer of cells, with a random-walk behaviour which acts to spread the endoderm over the yolk during early gastrulation stages (Fig. 1B) (Pézeron et al., 2008). By mid-gastrulation stages the endoderm begins to migrate towards the dorsal side of the embryo, converging to form two broad stripes on either side of the dorsal midline by early-somitogenesis stages; these stripes span the entire anterior-posterior length of the trunk of the embryo (Fig. 1C) (Mizoguchi et al., 2008).

Several lines of evidence demonstrate that the mesoderm directly regulates the migration of the endoderm to the dorsal midline during mid-gastrulation stages (Nair and Schilling, 2008; Mizoguchi et al., 2008; Pézeron et al., 2008). The overlying mesodermal and ectodermal cells have a directed migration towards the dorsal midline during gastrulation stages. The active and directional migration of these two cell types is demonstrated by the polarisation of their microtubule organising centres (MTOC) towards the midline (Sepich et al., 2011). The endoderm is still migrating to the dorsal midline during early somitogenesis stages (Mizoguchi et al., 2008) but it is unclear if the endoderm also develops a polarised MTOC. In this study we have investigated the state of endodermal polarisation during migration to the dorsal midline.

The morphogenic movements of the endoderm during somitogenesis stages have not yet been described in detail (Fig. 1E). What is apparent is that at some point the two stripes of single-layered endodermal cells become the single midline mesenchymal endodermal rod that has been observed at 24 h post fertilisation (hpf) (Fig. 1F) (Field et al., 2003b; Ober et al., 2003). To examine the movement of the endodermal cells during these stages we utilised the endodermal reporter line Tg(*sox17:EGFP*), which allows the visualisation of the endodermal cells up to 24 hpf. After formation of the mesenchymal endodermal rod, the endoderm undergoes epithelialisation to form a gut tube, and then organogenesis generates the gastrointestinal tract, liver, pancreas, swim bladder, and gall bladder (Ng et al., 2005; Field et al., 2003a,b).

The planar cell polarity (PCP) signalling pathway, also known as the non-canonical Wnt signalling pathway, is most well known for regulating the polarity of epithelial sheets perpendicular to the apical-basal axis (Mlodzik, 1999). PCP signalling was first identified in *Drosophila* (Gubb and Garcia-Bellido, 1982) in which it controls the orientation of the cuticular hairs in relation to neighbouring cells. PCP has been subsequently demonstrated to play a role in the polarisation of a large array of animal structures including left-right axis determination, orientated cell division of intestinal cells, left-right axis determination, hair follicle orientation, and the directionality of inner ear sensory hair bundles in mice (Fanto and McNeill, 2004; Mlodzik, 1999; Djiane et al., 2005; Lopez-Schier and Hudspeth, 2006; Devenport and Fuchs, 2008; Heydeck et al., 2009; Karner et al., 2009; Ravni et al., 2009; Antic et al., 2010; Oteiza et al., 2010; Wen et al., 2010; Matsuyama et al., 2009). PCP signalling has also been shown to play a major role in the regulation of mesenchymal cell behaviour during gastrulation and convergence and extension (C&E) in vertebrates (Matsui et al., 2005; Tada and Smith, 2000; Wallingford et al., 2000; Darken et al., 2002; Goto and Keller, 2002; Jessen et al., 2002; Ulrich et al., 2003; Veeman et al., 2003; Miyagi et al., 2004; Goto et al., 2005; Carreira-Barbosa et al., 2009; Vervenne et al., 2008; Dohn et al., 2013; Jenny et al., 2003; Tada et al., 2002). Much of the information gained so far about zebrafish PCP comes from studies of mesodermal C&E and neuroepithelial development (Carreira-Barbosa et al., 2003; Carreira-Barbosa et al., 2009; Ciruna et al., 2006; Dohn et al., 2013; Goto et al., 2005; Gros et al., 2009; Guirao et al., 2010; Heisenberg et al., 2000; Jessen et al., 2002; Kilian et al., 2003; Mapp et al., 2010; Marlow et al., 2004; Simons and Mlodzik, 2008; Tada and Kai, 2009; Topczewski et al., 2001; Veeman et al., 2003; Vervenne et al., 2008; Wada et al., 2005). PCP signalling is known to regulate C&E movements of mesodermal and ectodermal tissues during zebrafish gastrulation (Topczewski et al., 2001; Jessen et al., 2002; Marlow et al., 1998). However, how mesenchymal and epithelial cells use the specific PCP pathway components is still being elucidated. Loss of either of the core PCP signalling components, *vangl2* or *gpc4*, results in a wider and shorter body axis due to a reduction in C&E movements. *vangl2* and *gpc4* have non-redundant roles in regulating PCP signalling as *vangl2/gpc4* double mutants display an additive reduction in C&E (Marlow et al., 1998). *vangl2* and *gpc4* are both required for the polarisation of the MTOC in mesodermal and ectodermal cells (Sepich et al., 2011). MTOC polarisation is an accepted marker of the polarisation of cells undergoing active directional migration (Goulimari et al., 2008). Although disruption of PCP signalling appears to affect the C&E movements of all tissue layers during gastrulation stages (Marlow et al., 1998; Pézeron et al., 2008), a role for PCP signalling in the endoderm has not been examined; therefore, we investigated whether PCP signalling regulates either endodermal convergence to the dorsal midline, or formation of the endodermal rod.

In this study we have characterised the midline movement of the endodermal cells during somitogenesis stages. We have defined the process whereby endodermal cells leave the monolayer and form a mesenchymal rod midline aggregation. We investigated endodermal cell polarisation during somitogenesis stages, and identified that the endoderm does not generate a polarised cell state during migration to the dorsal midline. We have investigated PCP signalling in the endoderm during somitogenesis stages, and identified that it appears to be dispensable during early somitogenesis stages. Our results also propose the presence of a non-autonomous signal that regulates endodermal cells leaving the monolayer.

## RESULTS

### PCP mutants have disrupted endoderm morphogenesis

**Fig. 2.**
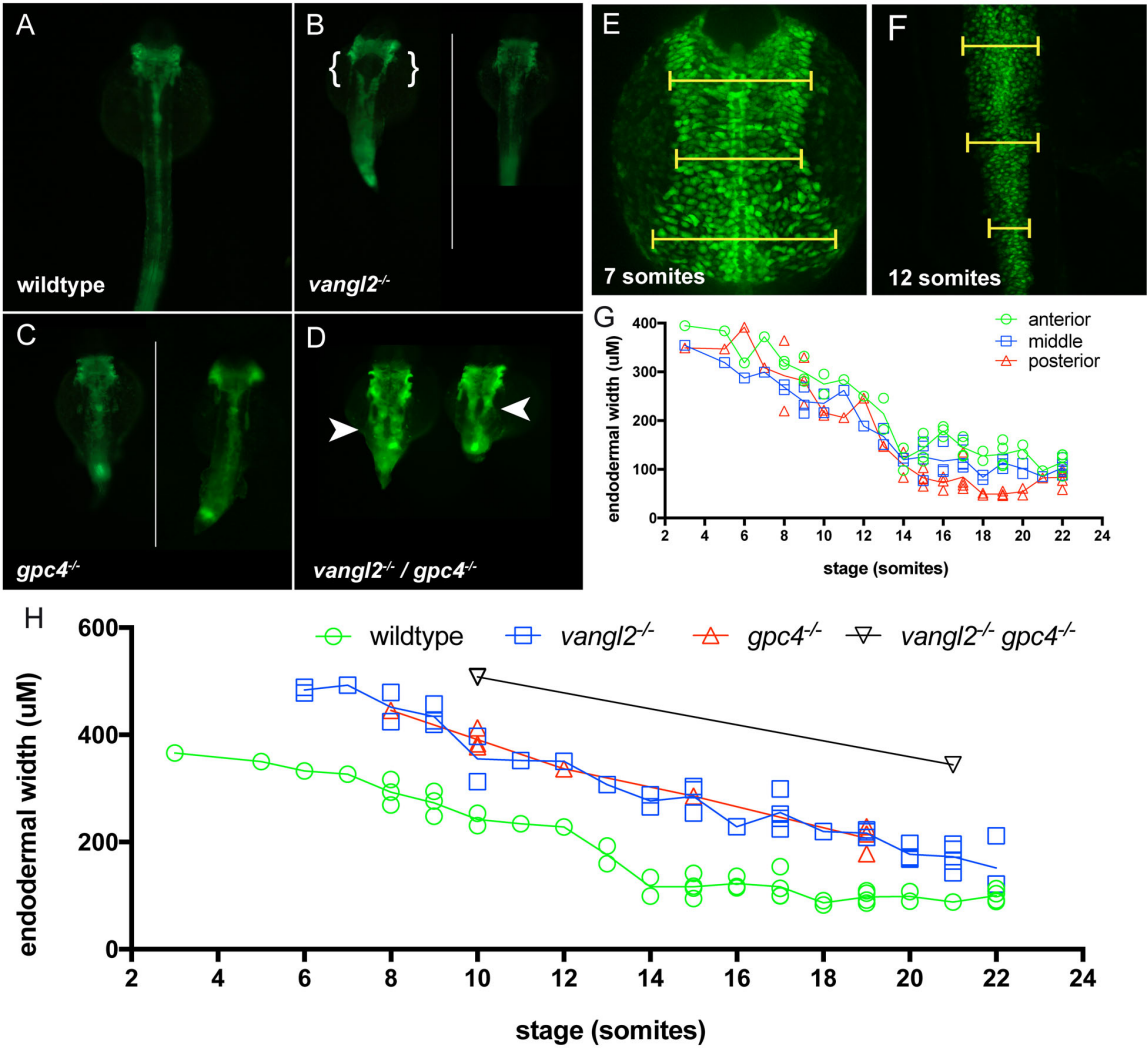
**Loss of PCP signalling results in disrupted endoderm morphology during development.** (A-D) Fluorescent dissecting microscope images of endoderm morphology at 24 hpf. All views are dorsal. (A) Wild-type and PCP homozygous mutant embryos (B) vangl2, (C) gpc4, and (D) vangl2l/gpc4 double mutants in the Tg(sox17:EGFP) background. Endoderm morphology in PCP mutants (B, C and D) is disorganised and wider than in wild-type (A). Disruption of PCP signalling in the single mutants occasionally results in a splitting of the endoderm posterior to the pharyngeal endoderm as indicated by the brackets in B. vangl2/gpc4 double mutant embryos always have a splitting of the endoderm posterior to the pharyngeal endoderm as indicated by the arrow heads in D. (E-F) Confocal projections of 7 somite (E) and 12 somite (F) wild-type Tg(sox17:EGFP) embryos. Dorsal views. Yellow bars indicate positions of the three width measurements. (G) The rate of change of the width of the endoderm, measured at anterior, middle, and posterior regions of the trunk endoderm, migrate with the same overall rate towards the dorsal midline during somitogenesis stages. (H) The endoderm is wider in homozygote vangl2 and gpc4 mutants and vangl2/gpc4 double mutant lines, although dorsal migration does still take place (n = 1-5 embryos per stage, per genotype). White lines in panel C and E divide grouped individual embryos.

The PCP mutants *vangl2* and *gpc4* have reduced convergence and extension of mesodermal and ectodermal tissues, resulting in a shorter wider embryo during somitogenesis stages (Topczewski et al., 2001; Jessen et al., 2002; Marlow et al., 1998). To investigate if the endoderm was similarly affected in these mutants they were moved to the Tg(*sox17:EGFP*) endodermal reporter line. Using this background, it is clear that *vangl2* and *gpc4* mutants have disrupted endodermal morphology at 24 hpf (Fig. 2). *vangl2*/*gpc4* double mutants display a compounding effect with a drastically shorter and wider endodermal stripe relative to each respective single mutant (Fig. 2D). Although single mutants occasionally show a split in the endoderm with low penetrance (Fig. 2B bracket), this phenotype is always seen in the double *vangl2*/*gpc4* mutants (Fig. 2D arrowheads). This indicates that the loss of both *vangl2* and *gpc4* causes a greater defect than the loss of *vangl2* or *gpc4* alone, and demonstrates that these genes do not play redundant roles during endodermal morphogenesis.

The Tg(*sox17:EGFP*) line was used to examine the endodermal cell movements during somitogenesis stages. Interestingly, the movement was not confined to a short, fast movement, but was a sustained narrowing of the endodermal band at an even rate of 43.17 µm/h throughout the stages from 3-somites stage (11 hpf) to 18-somites stage (18 hpf), at which time the endoderm has essentially completed this movement (Fig. 2G). The earliest *vangl2* and *gpc4* mutants can be visually phenotyped is at 3 somites. At this stage, the endoderm of the *vangl2* and *gpc4* mutants is beginning the migration movement from a wider point than that of wild-type embryos, and despite a very similar migration rate of 41.83 and 41.63 µm/h, respectively, the endoderm was still distinctly wider than in wild-type embryos during all the somitogenesis stages (Fig. 2H). This resulted in a wider endoderm at the end of somitogenesis. The endoderm was wider again in the double mutants, both at the start and the end of the endodermal migration movement. These results suggest that PCP signalling may play a role in the morphogenesis of the band of endodermal cells, and so this warranted further investigation. One possibility is that the wider endoderm phenotype in the PCP mutants resulted from a loss of cellular polarity during convergence of the band of endodermal cells to the midline.

### Endodermal MTOC is not polarised during convergence

**Fig. 3.**
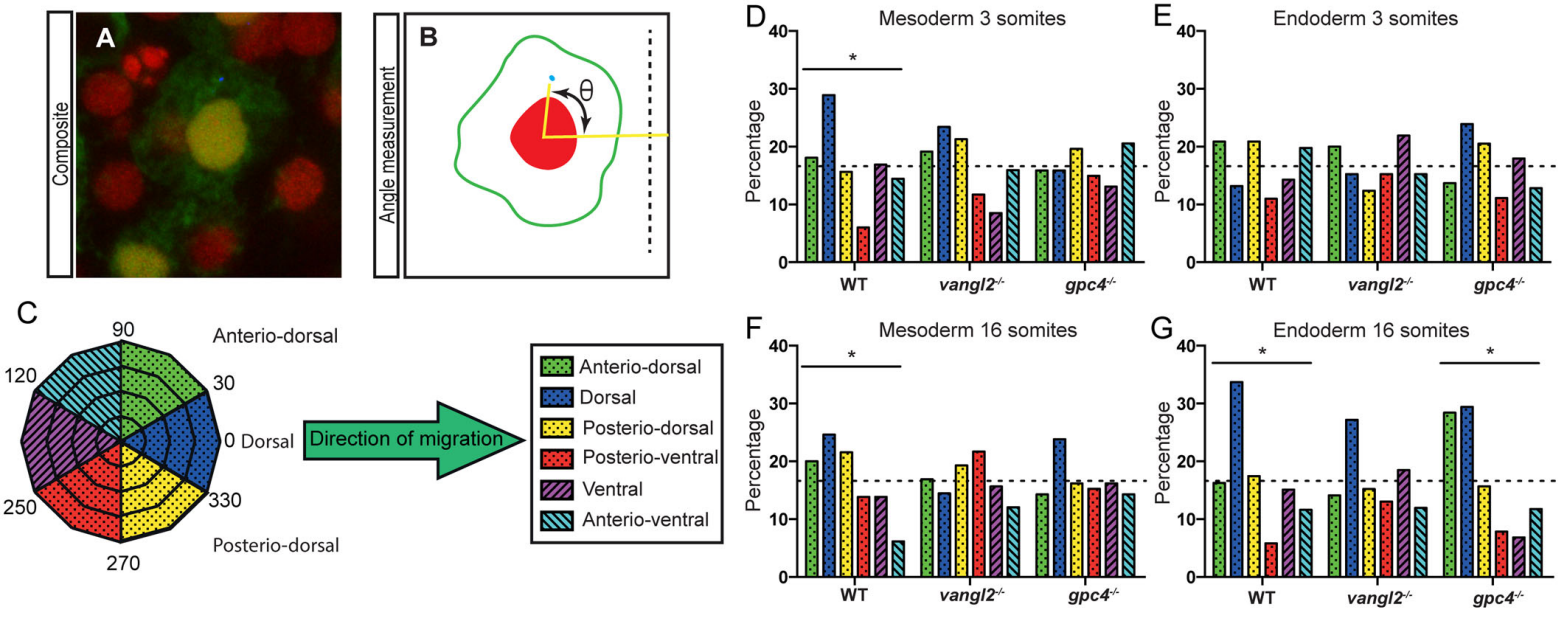
**MTOC distribution of mesoderm and endoderm during somitogenesis stages.** (A) Composite confocal micrograph demonstrating an EGFP-positive endoderm cell with red nucleus and blue MTOC. (B) Schematic angle measurement (θ) of the MTOC relative to the centre of the nucleus and the dorsal midline (dotted line). (C) Schematic highlighting the grouping of MTOC data into six direction groups. (D-G) Graphs showing the percentage of MTOC in each of the six segments. (D) Mesoderm 3 somites stage. (E) Endoderm 3 somites stage. (F) Mesoderm 16 somites stage. (G) Endoderm 16 somites stage. Dotted line indicates equal (randomised) mean value. Asterisk represents significant difference by χ squared test, *P≤0.05. See Table S1 for χ squared values testing for equal MTOC distributions over the six segments (null hypothesis is randomised data) and with wild type observed values as expected values (null hypothesis is data matching wild type). See Table S2 for cell number and embryo number analysed.

Previous studies have demonstrated that the mesodermal cells show a polarised MTOC during C&E stages. We investigated whether this polarisation is maintained into somitogenesis stages. We extended this study into endodermal cells, to determine whether they also generate a polarised MTOC during convergence to the midline, which would indicate that their movement to the midline constitutes an active migration. We determined the position of the MTOC relative to the nucleus in the endodermal and surrounding mesodermal cells, located at the midpoint of the anterior-posterior axis, using confocal microscopy of *TagBFP-Xcentrin* mRNA and *nuclear-RFP* mRNA injected embryos. Early- (3 somite, 11 hpf) and mid-somitogenesis (16 somites, 17 hpf) time points were chosen for analysis, as the endodermal cells are converging towards the dorsal midline at 3-somites stage (Mizoguchi et al., 2008), and by 16-somites stage they have predominantly finished their midline movement (Fig. 2H). MTOC polarisation was quantified by grouping angle measurements into six 60° segments relative to the direction of migration towards the dorsal midline (Fig. 3C). The results for each of the segments were graphed (Fig. 3D-G). A χ squared test was applied to identify MTOC polarisation in a specific direction compared to equal values across all segments (randomised) (Fig. 3D-G, dotted line; Tables S1 and S2). Using this method, we observed that the mesoderm maintained its MTOC polarisation towards the dorsal side of the embryos, from C&E stages into both early- and mid-somitogenesis stages (*P*=0.00611 and 0.0194, respectively) (Fig. 3D,F). PCP signalling has been shown to be essential for the maintenance of the MTOC polarisation in mesodermal and ectodermal cells at C&E stages as they migrate as individual cells (Sepich et al., 2011), and as an extension to this, we observed that disruption of PCP signalling also abolishes the dorsal orientation of the MTOC in the mesoderm during early- and mid-somitogenesis stages. In contrast to the mesodermal cells, the endodermal cells of wild-type embryos did not have a directionally polarised MTOC during movement to the midline at 3 somites (*P*=0.3353) (Fig. 3E). This indicates that endodermal cells are able to move to the midline during somitogenesis without a polarised MTOC, and therefore they are not undergoing an autonomous directional migration as isolated cells during these stages.

At mid-somitogenesis stages the endoderm develops a dorsally directed MTOC polarisation (*P*=0.0001) (Fig. 3G). The establishment of MTOC polarisation at this time point suggests that the endoderm is undergoing an active migration, and that this migration is directed towards the dorsal midline. As MTOC polarisation requires PCP signalling in the mesoderm and ectoderm, we hypothesised that MTOC polarisation might be lost in the *vangl2* and *gpc4* mutants. Although *vangl2* mutant embryos had an endodermal MTOC distribution that was different from wild type (*P*=0.0409), it was not statistically polarised (*P*=0.0923) (Fig. 3G), indicating that MTOC polarisation was indeed lost in *vangl2* mutants. Surprisingly, *gpc4* mutants were still able to generate a polarised MTOC in the endodermal cells during mid-somitogenesis stages (*P*=0.00001), despite losing MTOC polarity in the mesoderm (*P*=0.5652). The difference in results for *vangl2* and *gpc4* mutant embryos provides additional evidence that *vangl2* and *gpc4* have non-redundant functions during endoderm development.

### Endodermal cells begin leaving the monolayer at 11 somites and PCP signalling is dispensable for this process

**Fig. 4.**
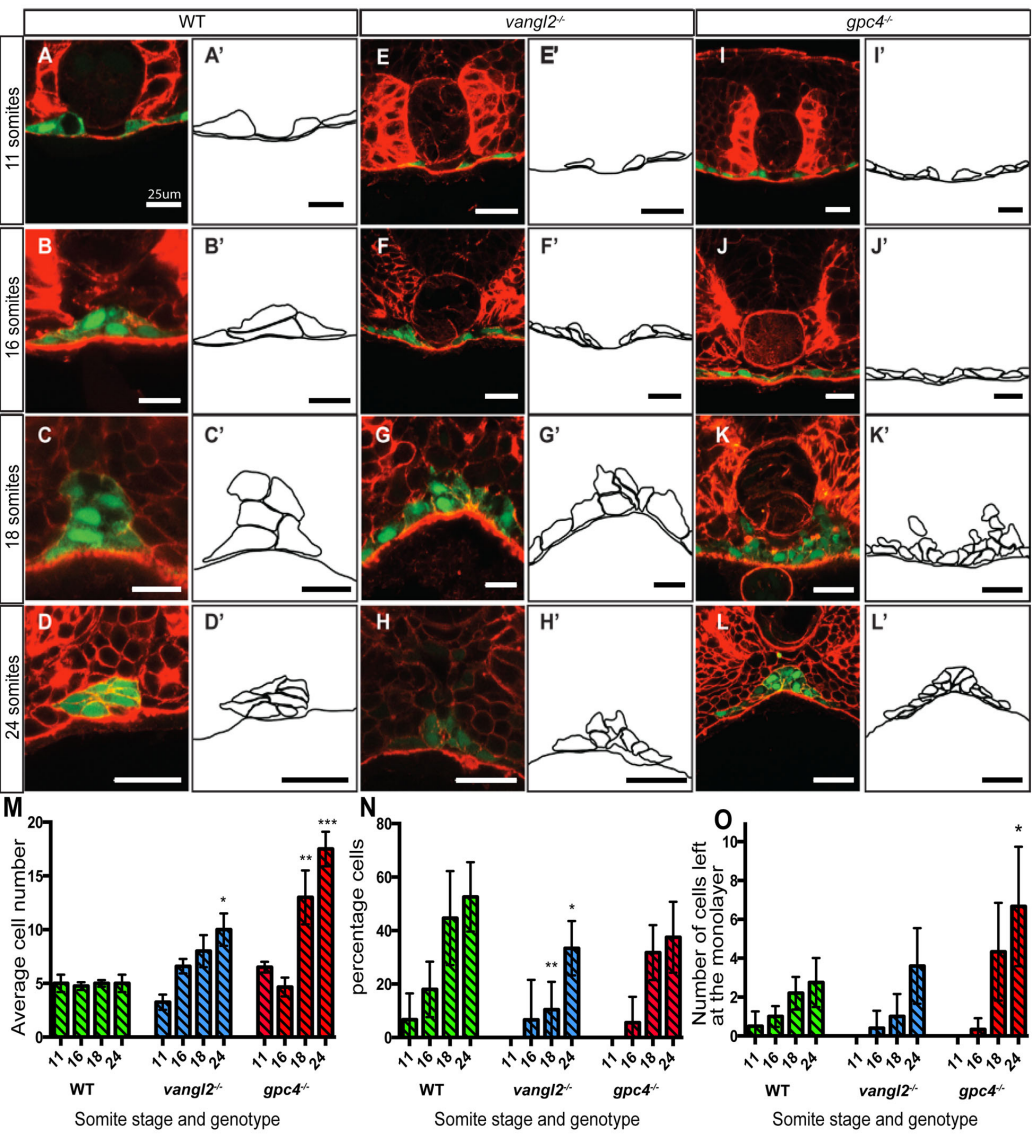
**Midline aggregation of endodermal cells in wild-type and PCP mutant lines.** (A-L). Confocal images of sections stained with Rhodamine-Phalloidin to show F-actin (red) at 11 somites, 16 somites, 18 somites, and 24 somites. Endoderm labelled in green by Tg(*sox17:EGFP*). Vibratome sections are in the region of the trunk endoderm to control for A-P position. (A′-L′) Schematic representations of the endoderm at each stage. (A-D) Wild-type embryos. (E-H) *vangl2^−/−^* embryos, and (I-L) *gpc4^−/−^* embryos, showing a wider endodermal region and an increase in endodermal cell number. Scale bar: 25 μm. (M) Mean cell number at the dorsal midline from 11 somites to 24 somites of wild-type (green bars), *vangl2*^−/−^ (blue bars), and *gpc4*^−/−^ (red bars) embryos. (N) Percentage of endodermal cells that have left the monolayer in wild-type (green bars), *vangl2*^−/−^ (blue bars), and *gpc4*^−/−^ (red bars) embryos. (O) Total number of endodermal cells that have left the monolayer in wild-type (green bars), *vangl2*^−/−^ (blue bars), and *gpc4*^−/−^ (red bars) embryos (*n*=2-8). Error bars represent s.e.m. Asterisk represents significant as determined by *t*-test, *P≤0.05, **P≤0.01, ***P≤0.001.

To understand the movements of endodermal cells during midline aggregation we examined confocal images of wild-type embryo cross-sections throughout somitogenesis (Fig. 4). The endodermal cells that have accumulated either side of the midline have been in a neat monolayer on the yolk membrane up to this point. We observed that they first left this monolayer at 11 somites (14.5 hpf). We defined this stage as the beginning of midline aggregation, the stage during which the cells aggregate together to form the midline rod. Endodermal cells continued to leave the monolayer at the dorsal midline from 11 somites onwards until formation of the mesenchymal rod at 24 hpf (Fig. 4A-D′).

We observed that endodermal cells had a polarised MTOC at 16 somites, and that this was lost in *vangl2* mutants (Fig. 3G), and we therefore hypothesised that disruption of PCP signalling would affect endodermal midline aggregation. The endodermal cells in *vangl2* and *gpc4* mutant embryos were delayed in leaving the monolayer compared to wild type, as they didn't begin to leave the monolayer until 16 to 18 somites (Fig. 4E-L′). It is interesting to note that in *vangl2* and *gpc4* mutants endodermal cells leaving the monolayer did so only in the region localised at the midline, and that cells at the lateral regions were never observed leaving the monolayer. Importantly, however, endodermal cells did still leave the monolayer and undergo midline aggregation in both *vangl2* and *gpc4* mutants by 24 hpf, and therefore it can be concluded that PCP signalling is not required for endodermal cells to leave the monolayer during midline aggregation, although its loss does cause a delay. These observations suggest that the cue that initiates cells to leave the monolayer may be influenced by the overall endodermal width.

The behaviour of endodermal cells at the midline could be altered by changes to the number of cells at that specific anterio-posterior position, and so we quantified the number of endodermal cells in each cross-section (Fig. 4M). Throughout midline aggregation it was observed that the number of mediolateral endodermal cells remained constant (∼5 cells per section) in wild-type embryos, but *vangl2* and *gpc4* mutants had an increase in the number of endodermal cells mediolaterally during midline aggregation. Mediolateral cell number increased significantly relative to wild-type embryos at the stage when the tail would normally leave the yolk and begin to extend, a morphogenetic event that is severely reduced in *vangl2* and *gpc4* mutants. It is possible that the failure to extend the tail in these PCP mutants has a direct effect on the convergence of more anterior tissues. Although *vangl2* and *gpc4* mutants were wider and had more endodermal cells mediolaterally than wild-type embryos, the cells that left the monolayer were only positioned at the dorsal midline, under the notochord, rather than on the margins of the endodermal stripe (Fig. 4H′,L′). This observation suggests the presence of a regulatory cue, located at the dorsal midline, which determines the region of endoderm that can leave the monolayer.

## DISCUSSION

### Midline aggregation is a distinct phase of endoderm morphogenesis

Here, we have characterised a phase of endoderm morphogenesis termed midline aggregation, in which endodermal cells that have accumulated either side of the midline begin to leave the monolayer and form a mesenchymal endodermal rod. Midline aggregation begins when endodermal cells start leaving the monolayer at 11 somites (14.5 hpf). Midline aggregation can be defined as a distinct phase of endoderm morphogenesis as it is the first point since specification that endodermal cells are no longer present as a monolayer or in contact with the underlying yolk membrane. In addition, it is during midline aggregation that the endoderm first develops a polarised cell state. Prior to this stage the endodermal cells do not have a polarised MTOC, which we reasoned indicates that the movement towards the midline, up until 11 somites, is a continuation of C&E movements in which the movement of the endoderm is directed by mesodermal cells, followed by midline aggregation from 11 somites to 21 hpf. Midline aggregation is considered complete when 50% of endodermal cells have left contact with the yolk syncytial layer (YSL) and formed a mesenchymal rod at 24 somites (21 hpf).

### Endodermal cell migration is not active, but midline aggregation is

We identified that the endoderm does not have a polarised MTOC during migration to the dorsal midline at early somitogenesis stages. The lack of MTOC polarisation indicates that the endoderm is not undergoing an active migration towards the dorsal midline at these stages. This is in contrast to the surrounding mesoderm, which maintains its polarised MTOC from C&E stages into early somitogenesis stages. Previous observations in the literature indicate the migration of endodermal cells towards the midline is influenced by the migration of the overlying mesoderm (Pézeron et al., 2008), either by a physical tether (Nair and Schilling, 2008), or a chemokine gradient (Mizoguchi et al., 2008), and our results are consistent with these models. In addition, our data does not distinguish between these models.

The generation of a polarised MTOC in the endodermal cells during midline aggregation stages, while endodermal cells are leaving the monolayer, indicates a coordinated and active morphogenetic movement in contrast to a passive movement controlled by the surrounding mesoderm. At these stages the mesoderm overlying the endoderm is undergoing dramatic morphogenetic movements in the process of generating somites (Hollway et al., 2007), and therefore would no longer be interacting with and influencing the underlying endoderm as it did during gastrulation stages.

### PCP signalling appears to not be required cell autonomously for these stages of endoderm development

Our data suggests that the endodermal defects observed during migration to the midline and midline aggregation in *vangl2* and *gpc4* mutants most likely result from PCP signalling being required for overall embryo morphology, rather than resulting from a specific endoderm-autonomous requirement for PCP signalling. Evidence for this comes from two observations. Firstly, our data indicate that the movement of the endodermal cells to the midline at early somitogenesis stages is not an active migration that requires polarised MTOCs. Endodermal cells at these stages do not generate a polarised MTOC, a process that is dependent on PCP signalling, and is required for independent or signal-directed movement. This therefore indicates that PCP is not required autonomously in the endoderm for cell movements that are dependent on a polarised MTOC. However, we also cannot exclude the possibility that PCP signalling is required in the endoderm for other aspects of cellular behaviour that control migration. Secondly, endodermal cells still undergo midline aggregation and leave the monolayer in *vangl2* and *gpc4* mutants despite the endoderm being wider, demonstrating that PCP signalling is not required for this process. Therefore, we propose that PCP signalling is not required autonomously in the endoderm during somitogenesis stages, but it is required instead to maintain the overall embryo topology that is required for correct endoderm morphogenesis. For example, our results show that endodermal cell number during midline aggregation in *vangl2* and *gpc4* mutants is different to wild type. This is possibly due to a combination of the shortening of the embryo, distributing the same number of endodermal cells along a shortened A-P length, and the failure of the tail to extend, preventing the thinning of the endoderm cells by convergence.

Further evidence for the overall embryo shape regulating midline aggregation comes from the observation that the width of the endoderm in *vangl2* and *gpc4* mutants when it does begin to leave the monolayer at 16 somites is comparable to the width of the endoderm in wild-type embryos when they begin leaving the monolayer at 11 somites (Fig. 1H). Together these data suggest that the overall width of either the endoderm or the embryo as a whole is a controlling factor determining the timing at which endodermal cells begin midline aggregation.

### A localised non-autonomous signal controls the region of endodermal cells leaving the monolayer during midline aggregation

Endodermal cells can be seen stacking up at either side of the dorsal midline from early somitogenesis stages (Mizoguchi et al., 2008). It is likely they are stacked up against the notochord. Yet we observed the two stripes either side of the midline do not contact each other or begin to leave the monolayer until 11 somites. This observation indicates that there is a specific signal that determines when the endoderm can initiate midline aggregation. In light of our observations we propose that there is a non-autonomous signal originating from the surrounding mesoderm that induces the endoderm to leave the monolayer. Reasoning for this hypothesis is as follows: if a signal autonomous to the endoderm dictates when cells leave the monolayer, cells would leave the monolayer along the entire width of the endodermal stripe, even in cases where the stripe is unnaturally widened. However, we observed when there is increased endodermal width and cell number, the cells leave the monolayer only in the region localised to the midline. Endodermal cells at the lateral regions were never observed leaving the monolayer. It seems most likely that the signalling cue to initiate endodermal cells to leave the monolayer is not autonomous to the endoderm, but instead originates from the surrounding mesoderm, specifically in the region of the dorsal midline. It is quite common for morphogenetic events to be triggered by external factors. Indeed, a similar situation has been demonstrated at later stages during liver organogenesis, where signals from the surrounding mesodermal tissues are required for correct endodermal morphogenesis (Niu et al., 2010).

### Conclusion

In this study we investigated the MTOC polarisation state of the endoderm as it migrates to the midline during somitogenesis stages, and identified that the endoderm is not actively migrating during this movement. Here we present data characterising and defining the morphogenic movements of the endoderm during midline aggregation; a developmental process whereby the two monolayer stripes of endodermal cells present on either side of the midline at somitogenesis stages coalesce to generate a single mesenchymal endodermal rod. Here we present the hypothesis that a non-autonomous signal from the surrounding mesoderm regulates the leaving of the monolayer by the endodermal cells during formation of the mesenchymal endodermal rod.

## MATERIALS AND METHODS

### Animal husbandry and zebrafish strains

Zebrafish strains used in this study were housed in either the research aquarium of Dr H. Verkade (School of Biological Sciences, Monash University) or the Australian Regenerative Medicine Institute (ARMI) FishCore aquarium at Monash University using standard husbandry practices. All experiments were approved by the Monash University Animal Ethics Committee. Mutant alleles of *vangl2/trilobite* (*tri^m209^* ZFIN ID: ZDB-ALT-980203-534) (Solnica-Krezel et al., 1996) and *glypican 4/knypek* (*kny^hi1688^* ZFIN ID: ZDB-ALT-020426-6) (Golling et al., 2002) were used in the background of the Tg(*sox17:EGFP*) reporter line [Tg(*-0.5sox17:EGFP*) ZFIN ID: ZDB-TGCONSTRCT-080714-1] (Mizoguchi et al., 2006).

### mRNA injection

mRNA was transcribed from linearized plasmid from SP6 RNA polymerase using the mMessage mMachine Kit (Life Technologies). Fertilised one-cell stage embryos were microinjected with ∼2 nl of synthetic mRNA into the yolk cell.

### Vibratome sectioning of zebrafish embryos

Embryos were fixed in 4% PFA, then embedded in 4% low melting point agarose. 200 μM sections were taken using a Leica VT1200S vibratome. Sections were either directly imaged on a confocal microscope or stained before imaging. F-actin was stained with Rhodamine-Phalloidin (Life Technologies) as per manufactures recommendations.

### Endodermal width measurements

Endodermal width measurements were taken from both live and fixed embryos. Measurements were taken at three locations along the anterior-posterior axis; directly posterior to the pharyngeal endoderm (anterior), the middle of the trunk endoderm (middle), and at the widest point at the posterior end of the trunk endoderm (posterior). At stages where all three regions could not be imaged simultaneously in live embryos, embryos were fixed and flat-mounted prior to confocal imaging.

### Microscopy

Low power images were collected using an Olympus SZX16 equipped with an Olympus CC-12 camera. Confocal images were taken on either a Leica SP5 confocal microscope or a Nikon C1 upright confocal microscope with an immersion lens (either 20× Fluor 0.5 NA working distance 2.0 mm or 40× Fluor 0.8 NA working distance 2.0 mm). The brightness and contrast of images were adjusted, and images were imported into Photoshop CS3 (Adobe) for orientation. Figure preparation was preformed using Illustrator CS3 (Adobe).

### MTOC angle measurements

Embryos were fixed and flat mounted prior to confocal imaging. MTOC measurements were taken from a subset of endodermal cells located at the midpoint along the anterior-posterior axis of the endoderm. MTOC angle measurements relative to the nucleus and the direction of migration were calculated using Fiji imaging software (Schindelin et al., 2012).

### Statistics

Quantitative data derived from at least three independent experiments; descriptive statistics are mean±s.e.m. of data for *n* individuals or *n* independent experiments. Microsoft Excel 2007 was used χ squared statistics, *P*<0.05 was used to determine a statistically significant difference. GraphPad prism6 was used for unpaired two-tailed Student *t*-tests normally distributed continuous variables. *P* values are as follows *P≤0.05, **P≤0.01, ***P≤0.001.
